# A simulation study of cooperative and autonomous vehicles (CAV) considering courtesy, ethics, and fairness

**DOI:** 10.1371/journal.pone.0283649

**Published:** 2023-05-03

**Authors:** Liming Jiang, Yuanchang Xie, Nicholas G. Evans

**Affiliations:** 1 Department of Civil and Environmental Engineering, University of Massachusetts Lowell, Lowell, Massachusetts, United States of America; 2 Department of Philosophy, University of Massachusetts Lowell, Lowell, Massachusetts, United States of America; University of Cincinnati, UNITED STATES

## Abstract

Autonomous vehicles (AV) can be programmed to act cooperatively. Previous research on cooperative and autonomous vehicles (CAV) suggests they can substantially improve traffic system operations in terms of mobility and safety. However, these studies do not explicitly take each vehicle’s potential gain/loss into consideration and ignore their individual levels of willingness to cooperate. They do not account for ethics and fairness either. In this study, several cooperation/courtesy strategies are proposed to address the above issues. These strategies are grouped into two categories based on non-instrumental and instrumental principles. Non-instrumental strategies make courtesy/cooperation decisions based on some courtesy proxies and a user-specified courtesy level, while instrumental strategies are based only on courtesy proxies related to local traffic performance. Also, a new CAV behavior modeling framework is proposed based on our previous work on cooperative car-following and merging (CCM) control. With such a framework, the proposed courtesy strategies can be easily implemented. The proposed framework and courtesy strategies are coded in SUMO microscopic traffic simulator. They are evaluated considering different levels of traffic demand on a freeway corridor consisting of a work zone and three weaving areas of different types. Interesting findings are drawn from the simulation results, one of which is that the instrumental Local Utilitarianism strategy performs the best in terms of mobility, safety, and fairness. In the future, auction-based strategies can be considered to model how CAV make decisions.

## 1. Introduction

Recent research on Autonomous vehicles (AV) focuses on training them to make safe and efficient maneuvers from the viewpoint of individual vehicles. When a group of AV interact in the real world, even if they all make the “*best*” decisions individually, these interactions may not lead to a system-optimal state, similar to what the well-known Wardrop’s first and second principles describe. This research targets an important emerging research area in AV: cooperation among AV. It is possible to programing cooperation and courtesy into AV control algorithms, while we cannot do this to human-driven vehicles (HDV). This study focuses on two questions: (*1*) how to design ethical AV control algorithms that consider cooperative and courteous behaviors, and (*2*) how will such behaviors affect traffic system operations?

The importance of cooperation could be illustrated by the difference between Adaptive Cruise Control (ACC) and Cooperative Adaptive Cruise Control (CACC). ACC and CACC are typical examples of level-1 autonomy [1] and they both automate vehicle longitudinal control. ACC adapts vehicle velocity to ensure a safe following distance when there is a preceding vehicle. CACC is built on ACC. It further adopts wireless inter-vehicle communications for sharing additional information (e.g., acceleration rate) among two or more vehicles (not just the immediately preceding vehicle), which allows for precise longitudinal control of the following vehicle. Both analytical [2–4] and reinforcement learning [5, 6] methods have been proposed for CACC. In CACC, cooperation comes from the willingness of preceding vehicle(s) to share information. Such information can help the following vehicle reduce speed and acceleration oscillations [5], stabilize stop-and-go shockwave [2], and lead to significant safety benefits [7].

In addition to longitudinal control, cooperative lane change has been investigated in several studies. Ren et al. [8] developed a rule-based cooperative merge control strategy that substantially improves work zone mobility and safety compared to the widely used early merge and late merge. Wang and Chan [9] proposed a reinforcement-learning based vehicle agent that can generate safe, smooth and timely merging maneuvers. Existing studies on cooperative lane change could be grouped into three categories: highway ramp operations [9–13], work zone merge control [8, 14], and discretionary lane-changing decision [15, 16]. All these studies suggested that cooperation among AV plays an important role in improving traffic operations.

Existing methods for modeling cooperation among AV are inflexible, requiring AV to follow a pre-defined control strategy designed for maximizing system performance (e.g., safety and mobility). This cannot accommodate individual variation in user needs, for example an AV passenger in an urgent situation, such as when lawyer Joshua Neally was driven by his Tesla to a hospital while suffering a pulmonary embolism [17]. Alternately, AV in these inflexible models may have to spend extra time and cooperate with other vehicles even if AV passengers are willing to financially pay for uncooperative behavior. Another issue with existing studies is that they do not quantitatively consider fairness or the utility gain (loss) to individual AV requesting (performing) courteous or cooperative maneuvers. The control algorithms are designed to optimize the performance of all AV (i.e., the system). This may lead to systematic *discrimination against certain AV*: e.g., in a work zone AV in the open lane may be required to yield to vehicles in the closed lane to minimize total travel time, but only by disproportionately increasing the travel time of AV in the open lane [8, 14].

This study aims to address these concerns by incorporating efficiency, safety, cooperation, fairness, and ethics into modeling the *courteous* behavior of AV. The main contributions of this study are summarized as follows:

1. Based on our previous work on cooperative car-following and merging (CCM) control [18] for AV, a new behavior framework which incorporates courtesy evaluation module is proposed.
2. Courtesy and its derivatives are formally defined and quantified, which measure the willingness of AV to cooperate with others.
3. Different courtesy strategies for AV to make yield decisions are proposed and categorized into two major groups. One group consists of non-instrumental strategies, which take an individual view and require a courtesy level to be assigned to each AV. The other group is for instrumental courtesy strategies, where decisions are made by rules that are designed to achieve certain goals.
4. Microscopic simulation studies are conducted to evaluate each courtesy strategy in terms of mobility, safety, and fairness.

## 2. Background

The courtesy strategies proposed and evaluated in this study are all based on the CCM framework. To make this paper self-contained, the CCM control is briefly described in this section. Abbreviations used in this study are defined in Table 1.

**Table 1:** Abbreviations in this study.

SV	Subject vehicle, usually denoting the one that sends cut-in requests
FV	Front vehicle
LV	Lag vehicle
TFV	Target front vehicle
TLV	Target lag vehicle
RV	Vehicle that sends cut-in requests
TFG	Target front gap
TLG	Target lag gap
***Acc*_*free*_**	Acceleration for free driving
***Acc*_*CACC*_**	Acceleration for CACC
***Acc*_*CF*_**	Acceleration constraints for car following
***Acc*_*MP*_**	Acceleration constraints before merging point
***Acc*_*CCM*_**	Acceleration for CCM mode
***Acc*_*courtesy*_**	Acceleration for CAV that decides to yield
CF	Car following
MLC	Mandatory lane change
DLC	Discretionary lane change
Ego	Egoism courtesy strategy
Alt	Altruism courtesy strategy
CDE	Courtesy distribution expected (MIT survey)
CDM	Courtesy distribution moral (MIT survey)
LU	Local Utilitarianism courtesy strategy
LM	Local Maximin courtesy strategy
Ega	Egalitarianism courtesy strategy

**Fig 1:**
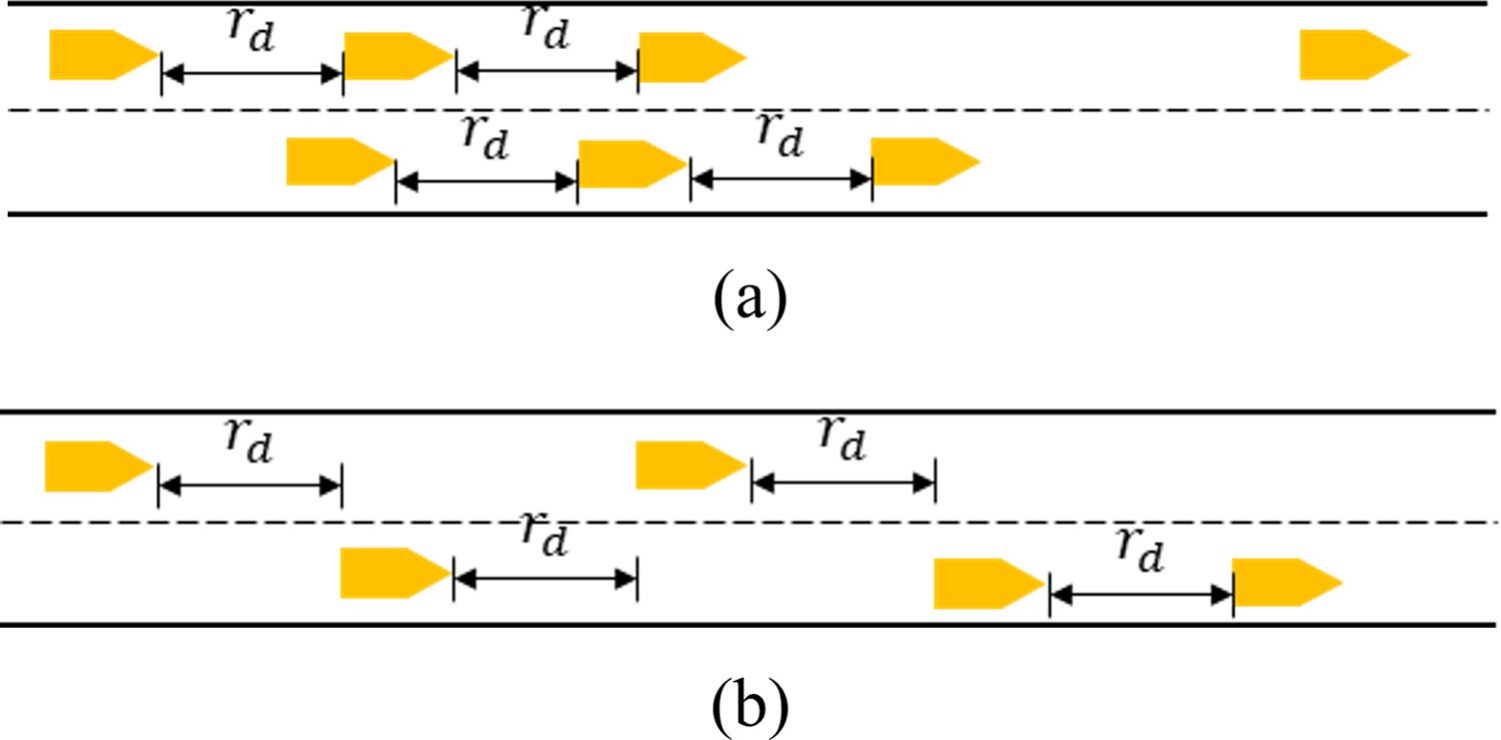
Comparison of Regular CACC/ACC (a) and CCM (b).

CCM control facilitates merge maneuvers at lane reduction points caused by accidents, work zones, etc. It is built on ACC and CACC. Instead of following the lead vehicle in the ***same*** lane, an AV controlled by CCM follows the nearest (in terms of longitudinal distance) downstream lead vehicle *regardless which lane it is in* as illustrated in Fig 1. This downstream lead vehicle is referred to as the generalized lead vehicle, or G-lead vehicle for short. With this straightforward extension of CACC/ACC, a CCM-controlled AV could keep a “safe” longitudinal distance with the G-lead vehicle and facilitate smooth merging maneuvers before the lane closure point.

With CCM control, an AV’s car-following behavior depends on whether its G-lead vehicle is equipped with an on-board device (OBD) to share real-time maneuver information. If the G-lead vehicle is equipped with OBD, the AV is assumed to have access to the G-lead vehicle’s acceleration, speed, and position information. Otherwise, the AV can only know the G-lead vehicle’s speed and position through its sensors (e.g., Lidar, camera). With CCM, the AV longitudinal behavior is described mathematically in Eq (1) below.

$$u_{2}(t)=\left\{ \begin{array}{l} k_{0}^{CACC}{\ddot{x}}_{1}(t)+k_{1}^{CACC}({\dot{x}}_{1}(t)-{\dot{x}}_{2}(t))+k_{2}^{CACC}(r(t)-\eta -\tau_{e}{\dot{x}}_{2}(t)),\;OBD=1\\ \;k_{1}^{ACC}({\dot{x}}_{1}(t)-{\dot{x}}_{2}(t))+k_{2}^{ACC}(r(t)-\eta -\tau_{e}{\dot{x}}_{2}(t)),\;OBD=0 \end{array}\right.$$ (1)

where,

*u*_2_(*t*) = acceleration of the following vehicle,

${\ddot{x}}_{1}(t)$ = acceleration of the G-lead vehicle,

${\dot{x}}_{1}(t)$ = speed of the G-lead vehicle,

${\dot{x}}_{2}(t)$ = speed of the following vehicle,

*r*(*t*) = current longitudinal distance between the G-lead and following vehicles,

*η* = jam distance,

*τ*_*e*_ = the desired effective time-gap, and

*k*_0_ = 1, *k*_1_>0, *k*_2_>0 = gains.

In Eq (1), OBD = 1 (0) indicates the G-lead vehicle is (is not) equipped with OBD. To differentiate from the traditional CACC, we refer to OBD = 1 as G-CACC mode. On the other hand (when OBD = 0), the AV will drive in the G-ACC mode. Just like the CACC and G-CACC modes, the only difference between ACC and G-ACC modes is that the AV will follow the G-lead vehicle, not the lead vehicle in the same lane.

This study assumes that all vehicles are AV and equipped with OBD. Therefore, for CCM control only G-CACC is applicable. With properly calibrated coefficients *k*_0_, *k*_1_, and *k*_2_ in Eq (1), a property of string stability [19] could be achieved. In this study, the parameters calibrated by Van Arem et al. [20] are used.

**Fig 2:**
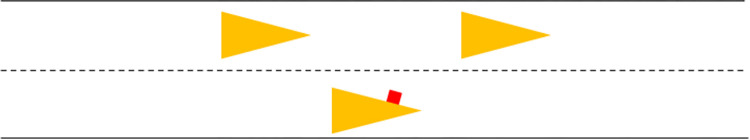
A lane-changing scenario.

With CCM, vehicles in different lanes cooperate with each other so that they are provided with safe target front gap (TFG) and target lag gap (TLG) before they reach the merging point. CCM requires all AV to follow the G-CACC mode all the time. However, this study relaxes this CCM assumption of full cooperation. As the example in Fig 2 shows, the ego vehicle in the right lane is trying to merge into the left lane. the ego vehicle would follow the target front vehicle (TFV) in G-CACC mode and send a cut-in request to the target lag vehicle (TLV) in the meantime. If TLV (also an AV) is courteous/cooperative enough to yield to the ego vehicle, it would approve the cut-in request and follow the ego vehicle in G-CACC mode (Otherwise, the TLV will not follow the G-CACC mode). When the TLG and TFG are sufficiently large (and other lane-changing criteria are met), the ego vehicle will change lane. More descriptions about the modified CCM strategy are given in the methodology section.

## 3. Methodology

### 3.1 Behavior model

**Fig 3:**
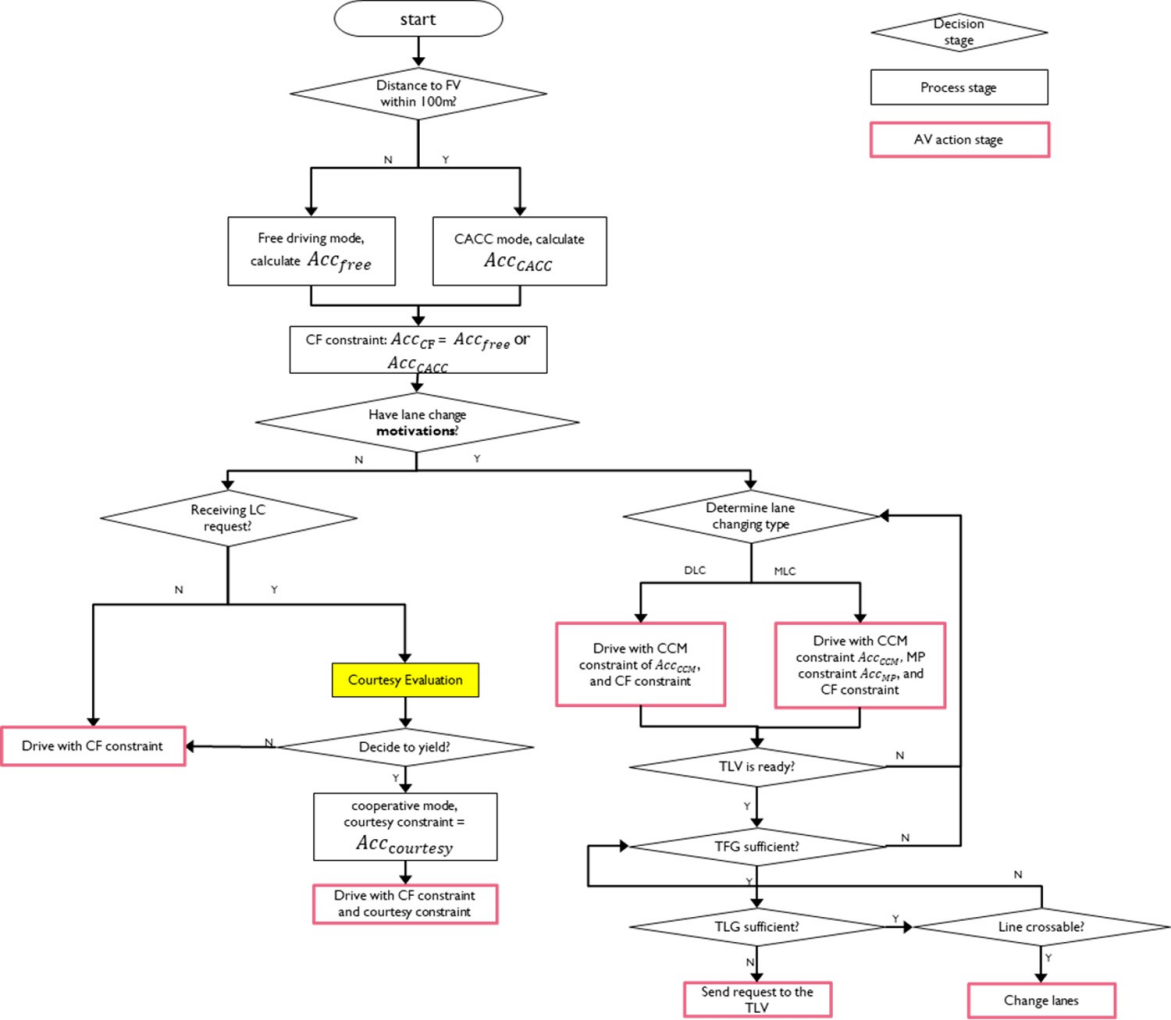
CAV behavior flowchart.

In this study, AV follow the behavior model outlined in Fig 3. The key component of the AV behavior model is courtesy evaluation, which is indicated by the yellow box in Fig 3. When an AV receives cut-in (e.g., lane change) requests for other vehicles, it will evaluate the current situation and decide whether to yield to others or not. More details about courtesy evaluation are given later in Section 3.2 Courtesy and Courtesy Strategies.

Fig 3 has three main types of stages: decision, process, and AV action. At every simulation step, an AV would begin with the “start” stage in Fig 3. After going through various subsequent stages, each AV will eventually arrive at least one AV action stage and execute the corresponding car-following and/or lane-changing actions. There are several important constraints on CAV acceleration. These constraints are detailed in the following four subsections.

#### 3.1.1 Car-following (CF)

AV longitudinal acceleration is denoted as *Acc*_*CF*_ and is constrained by the following factors (CF constraint). If there are no vehicles ahead of an AV within 100m, the AV will drive in free mode. The vehicle speed oscillates around an expected speed of 33 *m*/*s*. An acceleration of 2 *m*^2^/*s* (deceleration of -0.5 *m*^2^/*s*) would be adopted if the speed of the AV is less (greater) than the expected speed. Otherwise, the AV will drive in CACC mode and follow the formula with *OBD = 1* in Eq (1).

The AV would drive in the CF mode (either free or CACC) if it (1) does not have lane-changing motivation, (2) has not received any cut-in request, or (3) has received a cut-in request but decided not to yield. Under the CF mode, the G-lead vehicle in Eq (1) is simply the front vehicle (FV) in the same lane. Note that the CF constraint should also be enforced in some other AV action stages in Fig 3. For example, when an AV changes lane, it still needs to consider the CF constraint to avoid colliding with its FV.

#### 3.1.2 Mandatory lane change (MLC)

**Fig 4:**
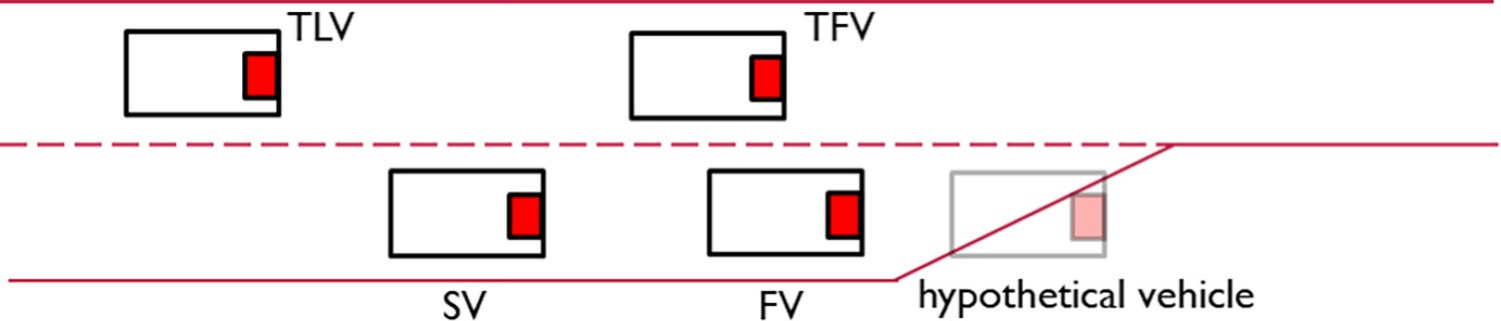
An MLC scenario.

As shown in Fig 4, an MLC involves four participants, which are the subject vehicle (SV), front vehicle (FV), target front vehicle (TFV), and target lag vehicle (TLV). *Acc*_*CF*_ is also used as a constraint here (i.e., the acceleration of SV should be equal or smaller than *Acc*_*CF*_) so that SV would not collide with its FV. Besides on ramps and lane reduction points, MLC can also happen before off ramps when vehicles have to change lanes in order to exit a highway.

For HDV, a vehicle’s lane-changing motivation typically increases as it approaches the merging point. In this study, AV follow “the earlier the better” principle and try to change lanes right after they enter the lane-change (LC) feasible region (e.g., acceleration lane for vehicles from the on-ramp to merge). However, successful lane changes are also subject to constraints such as sufficient TLG and TFG. Since an AV has to fully stop when reaching the merging point without being able to change lane, a deceleration constraint *Acc*_*MP*_ (*MP* stands for merging point) is introduced to make this happen. This deceleration is implemented by introducing a hypothetical stopped vehicle at the merging point in the closed lane, and the subject AV follows the CACC mode when it reacts to this “stopped vehicle”. The deceleration constraint *Acc*_*MP*_ is described in Eq (2) below and the only required input is the SV distance to the merging point (i.e., “stopped vehicle”).

$$Acc_{MP}(t)=k_{0}{\ddot{x}}_{1}(t)+k_{1}({\dot{x}}_{1}(t)-{\dot{x}}_{2}(t))+k_{2}(r(t)-\eta -\tau_{e}{\dot{x}}_{2}(t))$$ (2)

where ${\ddot{x}}_{1}(t)$ and ${\dot{x}}_{1}(t)$ are the acceleration and speed of the hypothetical stopped vehicle, respectively, and they are both set to 0. *r*(*t*) is the distance of the SV to the stopped vehicle. All other parameters are defined in the same way as in the original CACC formula Eq (1).

To create enough space in the target lane for the SV to merge into, the SV first executes CCM (with *Acc*_*CCM*_, as indicated in Eq (1)) to generate a safe gap to the TFV. At the same time, the SV would perform a feasibility check to see if the TLV is ready for the SV to merge into its lane. This check is done by calculating the forced deceleration of the TLV assuming the SV is already in the target lane. If the calculated deceleration is larger than the maximum deceleration allowed by the TLV, the feasibility check is considered failed. The forced deceleration is calculated following the G-CACC formula (OBD = 1) in Eq (1) except that the following vehicle is the TLV and the G-lead vehicle is the SV.

The feasibility check is to make sure the intended lane change will not cause trouble to the TLV. If the feasibility check is passed, the SV would further execute a gap acceptance check just like human drivers do to ensure safety from the SV perspective. The difference is that the SV would adopt smaller gaps than human drivers. The gap acceptance check is based on a certain threshold rather than probability. A gap would be accepted if it is larger than a critical time gap threshold. Both front and lag gaps need to be larger than the threshold. The front gap can be created by the CCM strategy. However, sometimes the lag gap (because of TLV) is not enough for the SV to change lane. In this case, the SV would send a LC request to the TLV for creating a larger lag gap. Upon receiving the request, the TLV would perform a courtesy evaluation and decide what to do next. More details on the courtesy evaluation are discussed later in this paper.

To conclude, the acceleration required for the SV due to MLC is governed by three constraints, which are *Acc*_*MP*_, *Acc*_*CCM*_, and *Acc*_*CF*_. The minimum of them will be chosen. For lane-changing maneuvers of the SV, they are based on feasibility check and gap acceptance check.

**3.1.3 Discretionary lane change (DLC).** The motivation of DLC is different from MLC. DLC is for pursuing higher speed or reducing travel time. In this study, AV DLC is modeled using utility. An AV would choose lanes based on their utilities. The utility of target lane *i* is defined as:

$$U_{lane_{i}}=MIN(v_{t},v_{lane_{i}})$$ (3)

where *v*_*t*_ is the speed of the target vehicle, and $v_{lane_{i}}$ is the average speed of target lane *i*. If the SV is currently in target lane *i*, the target vehicle is the SV’s FV. If the SV is not in target lane *i*, the target vehicle is the TFV. A utility difference threshold is defined so that the SV would not change lanes too often. In other words, DLC itself has an initial cost. To overcome this initial cost, the utility gain from changing lane has to be greater than a threshold of 5 *m*/*s*.

Once the utilities of each lane have been determined, the SV’s DLC decision can be made. What happens next for the SV is not much different from MLC. The SV can still send requests via vehicle-to-vehicle communications for a larger TLG. The only difference is that *Acc*_*MP*_ would not be considered as a constraint.

**3.1.4 Courtesy acceleration.** MLC and DLC are control logics from the perspective of the SV. In this subsection, courtesy acceleration (***Acc***_***courtesy***_) is introduced to model the behavior of the TLV. If a TLV decides to yield to another lane-changing vehicle, ***Acc***_***courtesy***_ is the corresponding acceleration needed. Again, ***Ac****c*_*courtesy*_ is determined following the CCM framework in Eq (1), in which the following vehicle is the vehicle behaving courteously (i.e., TLV) and the G-lead vehicle is the one sending the cut-in request.

### 3.2 Courtesy and courtesy strategies

Some important concepts need to be introduced before going forward. Courtesy may mean a number of things in social interactions, and might be characterized as either a behavior (e.g. opening a door for another is the courteous thing to do) or a disposition (i.e. one has the character traits of *being courteous*). Presumably, AV have no dispositions, so we rely on courtesy in the context of MLC/DLC as cooperating with another vehicle on the road when a lane change is necessary as part of the flow of traffic. From here we can define three important terms for our model: *courtesy level*, *courtesy proxy*, and c*ourtesy principles*. Courtesy level is a pre-determined attribute of AV, and it measures AV’s level of willingness to cooperate. *Courtesy proxy* is an environment-dependent variable, and it is utilized by AV to decide whether to cooperate or not. *Courtesy principles* (or *courtesy strategies*) are a group of principles and/or strategies taken by AV to achieve various goals.

Fig 3 shows how AV in this study would decide whether to cooperate (i.e., show courtesy—in the case of MLC/DLC, yield) or not when receiving Lane-Changing (LC) requests. In previous studies, this decision process is implicitly considered in AV’s behavior model [8, 9] whose disadvantages are discussed in the previous section. Here, courtesy is explicitly modeled by

1. following how human behave: AV with high courtesy levels would be more likely to yield in a given scenario, but AV can have different courtesy levels that determine their cooperation.
2. pursuing certain systematic goals. In other words, courtesy can be important for its own sake (i.e., as a principle of a good road system) or can be instrumental to some other good (e.g. to minimize congestion).

This paper provides a set of courtesy principles to guide courteous interactions among AV, using speed difference as a courtesy proxy. In future work this could be replaced by other proxies such as time to crash (as a proxy for safety) or required maximum deceleration rate (for passenger experience and safety). Noninstrumental principles have been considered elsewhere, albeit not for courtesy, but we set them aside here for simplicity’s sake [21]. The proposed courtesy strategies are categorized into two groups: non-instrumental (Egoism and Altruism) and instrumental (Local Utilitarianism, Local Maximin and Local Egalitarianism), and they are detailed in the rest of this section. The strategies chosen are popular in autonomous vehicles research that focuses on ethical decisions made by these vehicles. Utilitarianism is one of if not the most commonly considered moral system for autonomous vehicle decision-making [22], while there has been recent attention to maximin as a proxy for popular social contract theories of ethics [23, 24]. Egalitarianism is less studied but is seen as a proxy for a particular kind of fairness in interactions of vehicles in which the parameter in question is equalized, or the benefit distributed, between parties [25].

#### 3.2.1 Egoism

Under Egoism, Target Lag Vehicle (TLV) yields only when it would be in its own interest to do so–here, when it would not require the TLV to sacrifice too much of its own velocity. In this study, we use a *raw courtesy level* (RCL) to describe the maximum velocity loss the TLV will incur in the name of cooperating with other cars. For example, a TLV with a RCL of 5 m/s would yield in a scenario that requires it to decelerate by 3 m/s, since 3 m/s is less than the prespecified 5 m/s. The courtesy proxy (CP) used in Egoism is thus defined as the speed difference of the TLV before and after a lane change.


$${CP}_{Ego}=v_{TLV}^{before}-v_{TLV}^{after}$$ (4)

Courteous behavior of TLV would be triggered if *CP*_*Ego*_ is less than the courtesy level of the TLV as shown in Eq (5). Otherwise, the TLV will ignore the cut-in request and choose not to cooperate.


$$\mathrm{TLV}\;\mathrm{will}\;\mathrm{cooperate}=\left\{ \begin{array}{l} 1,\;if\;courtesy\;proxy\leq RCL\\ 0,\;if\;courtesy\;proxy>RCL \end{array}\right.$$ (5)

Raw courtesy level can vary for each AV, representing its individualized willingness to cooperate. As shown in Eq (6), it is derived from a standard courtesy level which ranges from 0 to 1.


$$\mathrm{RCL}=\mathrm{standard}\;\mathrm{courtesy}\;\mathrm{level}*v_{s}$$ (6)

Here, 0 means AV will never yield to others, and 1 means that AV will always cooperate if there is a request. *v*_*s*_ is the maximum speed of the subject vehicle (which usually is the speed limit on the road).

#### 3.2.2 Altruism

Under Altruism, TLV makes cooperation decisions from the perspective of the vehicle requesting courtesy (i.e., cut-in requests). In this case, TLV would yield if the utility gained by the SV requesting courtesy is less than what the TLV’s courtesy level allows. For example, TLV with a RCL of 5 m/s would not yield in a scenario where the SV could speed up from 5 m/s to 11 m/s after a lane change, since the speed gain is greater than the prespecified courtesy level (11 m/s– 5 m/s > 5 m/s).

The courtesy proxy for Altruism is defined in Eq (7), which is the speed difference of SV (i.e., the one sending cut-in request) before and after a lane change. As in Egoism, courteous behavior of TLV would be triggered if the courtesy proxy is less than TLV’s RCL as in Eq (5). The courtesy proxy for each AV under Altruism is also calculated based on a standard courtesy level as in Eq (6).


$${CP}_{Alt}=v_{SV}^{After}-v_{SV}^{Before}$$ (7)

#### 3.2.3 Local Utilitarianism (LU)

Local Utilitarianism strategy accounts for the utility gain of the SV attempting to change lane and the sacrifice (i.e., utility loss) to be made by the TLV. The TLV would yield to the SV if the sum of the utility resulting from a change is positive, accounting for the change in utility in both vehicles. It is called local utilitarianism because only two vehicles are involved and the immediate impacts (speed changes in this study) are considered in the decision process. However, the local decision may also benefit the operations of the entire traffic system. While considering more vehicles and the broader impacts (e.g., missing a downstream green light) of local cooperative behavior is desirable, it is very difficult to quantify and capture such effects given the complex relationship between individual vehicle behaviors and traffic system dynamics.


$${CP}_{LU}=(v_{TLV}^{after}-v_{TLV}^{before})+(v_{SV}^{after}-v_{SV}^{before})$$ (8)

One example of Local Utilitarianism (LU) is that TLV with a courtesy level of 5 m/s would yield in a scenario where the SV could accelerate from 1 m/s to 9 m/s after changing lane and the TLV would have to decelerate from 15 m/s to 9 m/s, since the sum of utility changes is positive (i.e., 9–1 + 9–15 = 2 > 0). The cooperative decision rules for TLV under LU are further defined in Eq (9).


$$\mathrm{TLV}\;\mathrm{will}\;\mathrm{cooperate}=\left\{ \begin{array}{c} 1,\;if\;{CP}_{LU}\geq 0\\ 0,\;if\;{CP}_{LU}<0 \end{array}\right.$$ (9)

#### 3.2.4 Local Maximin (LM)

The idea behind Local Maximin (LM) is that the worst off vehicle (here, the one with lower speed) should be made better off. In other words, LM aims to maximize the lower bound of the interaction, and like LU only considers the two vehicles in question. Based on this strategy, if the speeds of TLV before and after changing lane are 15 m/s and 7 m/s, respectively, and the corresponding speeds of SV are 3 m/s and 8 m/s, a courteous yield would be triggered, since the minimum speed is improved from 3 m/s (i.e., *Min*(15,3)) to 7 m/s (i.e., *Min*(7,8)).


$${CP}_{LM}=Min(v_{TLV}^{after},v_{SV}^{after})-Min(v_{TLV}^{before},v_{SV}^{before})$$ (10)

The LM decision rules for TLV are the same as those defined in Eq (9) except that *CP*_*LM*_ defined in Eq (10) is adopted instead of *CP*_*LU*_.

#### 3.2.5 Local Egalitarianism

Egalitarianism is related to LM: courteous behavior requires that the deviations between vehicle speeds from the real-time global average speed should be reduced. The courtesy proxy for Egalitarianism is defined in Eq (11).

$${CP}_{Ega}=[{\left(v_{TLV}^{before}-v_{G}\right)}^{2}+{\left(v_{SV}^{before}-v_{G}\right)}^{2}]-[{\left(v_{TLV}^{after}-v_{G}\right)}^{2}+{\left(v_{SV}^{after}-v_{G}\right)}^{2}]$$ (11)

where *v*_*G*_ is the real-time global average speed, which is the average speed of all vehicles in the network. The decision rules defined in Eq (9) still apply for this Egalitarianism courtesy strategy, but for *CP*_*Ega*_.

Assuming a scenario where the global average speed is 15 m/s; the speeds of TLV before and after changing lane are 16 m/s and 12 m/s, respectively; and the corresponding speeds of the SV are 5 m/s and 11 m/s. Since (16–15)^2^ + (5–15)^2^ > (12–15)^2^ + (11–15)^2^, a courteous behavior is triggered. The Egalitarianism strategy does not consider the courteous behavior’s impact on other vehicles except for TLV and SV, including the potential change in *v*_*G*_.

### 3.3. Simulation setup

**Fig 5:**
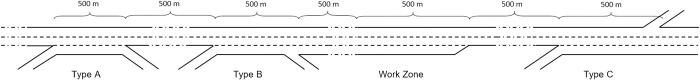
Simulation corridor.

To evaluate various courtesy strategies, a corridor comprising 4 critical segments is designed as in Fig 5. The four segments have different levels of traffic demand and require different types of lane-changing maneuvers. Two adjacent segments are separated by a 500-meter straight roadway segment without on/off-ramps. The various types of on- and off-ramps generate many opportunities for vehicles to behave courteously and to test the proposed courtesy strategies. From left to right in Fig 5, the four segments are Type A weaving area, Type B weaving area, work zone, and Type C weaving area, respectively. The layouts of these weaving areas are taken from the Highway Capacity Manual (HCM) [26].

To test the proposed courtesy strategies’ performance under different levels of traffic demand, three levels of traffic inputs (light, moderate, and heavy) are adopted, corresponding to different levels of service (LOS) described in the HCM [26]. Table 2 shows some sample service traffic volumes and the corresponding LOS taken from the HCM. In this study, light traffic represents LOS A, moderate traffic demand indicates LOS C, and heavy traffic input is for LOS E. LOS B and D are omitted for simplicity. If we use the traffic volumes directly from the HCM, upstream highway mainline traffic + onramp traffic–offramp traffic may not equal downstream mainline traffic, leading to imbalanced flows. To ensure flow conservation, minor adjustments (in the range of 25~125 veh/h/lane) are made to the traffic volumes corresponding to LOS A, C, and E in the HCM as in Table 2. In Table 2, numbers outside parentheses are directly from the HCM and those inside parentheses are final traffic volumes used in this study for light, moderate, and heavy traffic conditions. Note that only four volumes have been adjusted and are highlighted in bold font in Table 2. The same simulation parameters are used across different levels of traffic demand (e.g., free-flow speed: 120 km/s, volume ratio (weaving volume by non-weaving volume): 0.20, etc.)

**Table 2:** HCM example service volumes for freeway weaving segments.

Weaving Area Type	Service Volumes (veh/h) for LOS		
	A (light traffic)	C (moderate traffic)	E (heavy traffic)
**Type A**	1710 (1710)	3920 (**3970**)	5490 (**5640**)
**Type B**	1780 (1780)	4430 (**4280**)	6320 (**6170**)
**Type C**	1790 (1790)	4380 (4380)	6320 (6320)

The proposed AV behavior models as well as courtesy strategies are coded and simulated using SUMO [27] via the Traffic Control Interface (TraCI). Each simulation run is equivalent to one hour in the real world. The AV behaviors are updated at 2 Hz frequency, which means AV make decisions every 0.5 seconds. Such decisions include longitudinal control (accelerate or decelerate), lateral control (change lane or not), and communications with each other (sending and receiving lane-changing courtesy requests). Each combination of courtesy strategy and traffic demand (light, moderate and heavy) is simulated 10 times with different random seeds. The 10 simulation runs for each scenario allow us to calculate the means and standard deviations of performance metrics, which can be used to characterize how stable and reliable the simulation results are. As illustrated in Figs 7–11 below, the shaded areas are based on the means and standard deviations, showing the 95% confidence intervals for each strategy at each courtesy level.

For non-instrumental strategies, courtesy level can have significant impacts on the results. Therefore, the same strategy with different courtesy levels is regarded as distinctive strategies during the simulation. Table 3 summarizes the strategies simulated/evaluated in this study. For non-instrumental strategies (i.e., Egoism and Altruism), a courtesy level needs to be specified and all AV apply the same courtesy level throughout the simulation. Various courtesy levels have been tested. The test results suggest that the network performance is more sensitive to low courtesy levels. Therefore, an incremental interval of 0.02 is used when the courtesy level is between 0 and 0.2, while an interval of 0.1 is used for courtesy level between 0.2 and 1.

**Table 3:** Courtesy strategies tested.

Courtesy Strategy		Courtesy Level or Distribution
**Non-instrumental**	Egoism–Uniform	From 0 to 0.2 with 0.02 interval, and from 0.2 to 1 with 0.1 interval
	Altruism–Uniform	From 0 to 0.2 with 0.02 interval, and from 0.2 to 1 with 0.1 interval
	Egoism–Non-uniform / Distrubition	Courtesy distribution expected (CDE)
		Courtesy distribution moral (CDM)
	Altruism–Non-uniform / Distrubition	CDE
		CDM
**Instrumental**	Local Utilitarianism	N/A
	Local Maximin	N/A
	Egalitarianism	N/A

**Fig 6:**
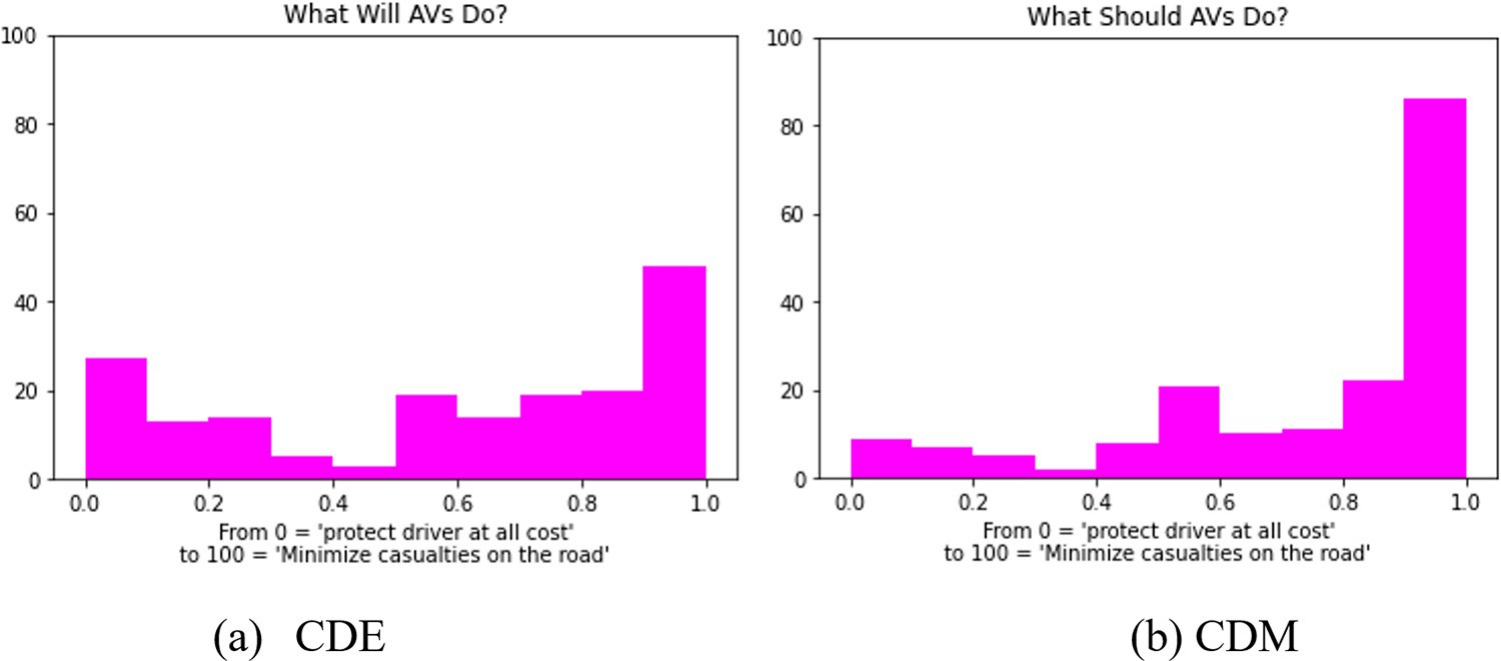
Two distributions from an MIT survey [22].

Heterogenous courtesy is also considered. In this case, AV courtesy levels could follow certain distributions. We opt to use two distributions described based on human preferences for altruistic or egoistic AV behavior conducted at the Massachusetts Institute of Technology (MIT) [22]. In this example, an AV has to either kill its passenger for saving ten pedestrians or kill the ten pedestrians to save the passenger. The choices provided range from 0 ‘protect passenger at all costs’ to 1 “minimize pedestrian casualties on the road”. The 182 survey respondents were asked this question in two distinctive ways: (1) what will AV do? and (2) what should AV do? These two ways resulted in two distributions shown in Fig 6. Considering the connection between the MIT survey and this study (i.e., should AV be concerned more about its passenger or other people), the two distributions are also adopted in this research to model AV courtesy level: Courtesy Distribution Expected (CDE) for “what will AV do”, and Courtesy Distribution Moral (CDM) for “what should AV do?”

### 3.4 Evaluation

The evaluation of courtesy strategies has been conducted at both network and individual segment levels in terms of mobility, safety, and fairness. At each time step, a vehicle could be in one of three possible states: courteous, lane-changing, and other. Being in *courteous state* indicates the SV is yielding to a lane-changing vehicle from adjacent lane. Being in *lane-changing (LC) state* means that the SV is sending LC request to its TLV in the target lane and managing to find a sufficient gap to cut in (the actual lane-changing behavior is assumed to occur instantaneously). All other situations are grouped into the *other state*. There is also an *all state* covering all those three nonoverlapping states, reflecting the average performance of all possible states. The performance measures are calculated for each state and all states combined.

Mobility is measured by global average speed. For the *i*^*th*^ state, the global average speed is obtained by:

$$v_{i}=\frac{\sum_{j}\sum_{t}r_{it}^{j}*v_{t}^{j}}{TS}$$ (12)

where *v*_*i*_ is the global average speed for the *i*^*th*^ kind of state. $v_{t}^{j}$ is the speed of vehicle *j* at time step *t*. $r_{it}^{j}=1$ indicates vehicle *j* is in state *i* at time step *t* and $r_{it}^{j}=0$ otherwise. *TS* is the total number of states for all vehicles, which is the sum of numbers of time steps experienced by all vehicles.

Safety is measured by Deceleration Rate to Avoid a Collision (DRAC). In this study, only the DRAC of TLV is considered when the SV is merging into the target lane. The DRAC for TLV is calculated as:

$$DRAC_{TLV}=0.5*{\left({v_{SV}-v}_{TLV}\right)}^{2}/s$$ (13)

where *v*_*SV*_ and *v*_*TLV*_ are the speeds of the SV and TLV (when the SV is changing lane), respectively. *s* is the corresponding space headway between the SV and TLV. *DRAC*_*TLV*_ measures how risky it is when a specific lane change occurs. A global DRAC is also calculated as the average of all *DRAC*_*TLV*_ observed during the entire simulation.

Fairness is also an important measure when courtesy and cooperation are considered [28, 29]. This study adopts Gini coefficient as a metric to measure system fairness. Two Gini coefficients are proposed: global Gini coefficient (G_GC) and categorical Gini coefficient (C_GC).

$$G\_GC=\frac{\sum_{i=1}^{n}\sum_{j=1}^{n}\left|v_{i}-v_{j}\right|}{2n^{2}\bar{v}}$$ (14)

where *n* is the total number of state speeds for all vehicles (if a vehicle stays in the network for 10 seconds, there will be 10 state speeds for it). *v*_*i*_ and *v*_*j*_ could be any state speed of all vehicles. $\bar{v}$ is the average state speed. *G*_*GC* essentially measures how imbalanced the distribution of all state speeds is. The more imbalanced it is, the larger *G*_*GC* becomes. *C*_*GC*, on the other hand, measures how imbalanced the mobility of a particular state is.

$$C\_GC=\frac{\sum_{k=1}^{3}\sum_{l=1}^{3}\left|V_{k}-V_{l}\right|}{2{*3}^{2}*\bar{V}}$$ (15)

where *V*_*k*_ (*k* = 1,2,3) is the average speed for a state (i.e., courteous state, LC state and other state). $\bar{V}$ is the average speed of *V*_1_, *V*_2_, and *V*_3_.

## 4. Analysis of results

### 4.1 Mobility

**Fig 7:**
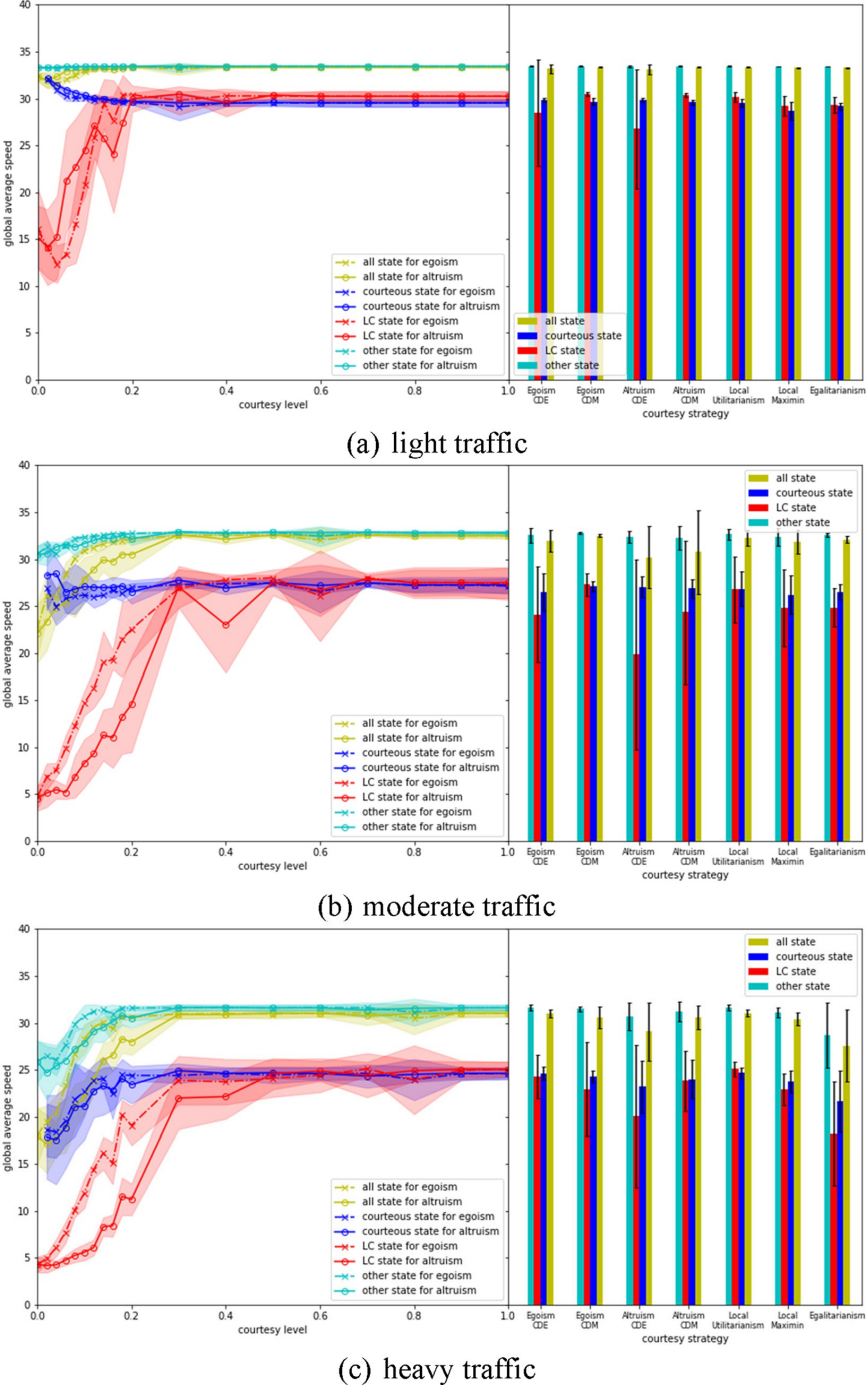
Evaluation of the mobility performance of different courtesy strategies.

Fig 7 shows how different courtesy strategies may affect traffic operations. The results for all states consider all vehicle states, including LC, courteous and other state. For Egoism and Altruism, courtesy level is modeled by two approaches. The first approach assumes all AV have the same courtesy level, which varies between 0 and 1 and leads to different simulation scenarios (see the left halves of each subplot in Fig 7). The second approach considers the two distributions generated by the MIT survey [22], and in each simulation run different AV can have different courtesy levels based on the CDE and CDM distributions. The results from the second approach are presented in the right halves of each subplot in Fig 7.

For Egoism and Altruism, the average speeds for all states in general increase monotonically as the courtesy level goes up regardless of traffic volumes—that is, as their tendency to cooperate increases according to Eqs (4), (5), and (7). This trend is also true for two component states, LC and other, except for some minor local fluctuations.

For courteous state the impacts of courtesy level on average speed also depend on traffic. Under low traffic volume, average speed changes with the inverse of courtesy. This trend is reversed, however, with high volume. *This suggests that the system benefits more from courteous behaviors when the traffic becomes more congested*. When Egoism and Altruism are compared on the same courtesy level, it could be concluded that Egoism results in better overall (for all states) mobility performance under medium and heavy traffic. Although Egoism performs slightly worse than Altruism under light traffic, the differences are much smaller than those under medium and heavy traffic. After decomposing all states into three component states, the superiority of Egoism compared to Altruism primarily comes from its advantage in LC state and other state.

For non-uniform courtesy, the mean and standard deviation of courtesy distribution from the MIT survey are 0.58 and 0.35 for CDE, and 0.75 and 0.30 for CDM. The system performance considering CDE and CDM (see the right halves of each subplot in Fig 7) are compared to those with uniform courtesy levels set to 0.58 and 0.75, respectively. It is found that the global average speeds of uniform courtesy for LC and courteous states are consistently higher than or at least equal to those of non-uniform courtesy. *It implies that for egoistic and altruistic courtesy strategies*, *a system with non-uniform courtesy may bring instability and thus cause system inefficiency*. A closer look at the results of individual simulation runs for CDE and CDM suggests that some of them (about *1~2* out of 10) have surprisingly low average speeds (under 5*m*/*s*) for LC state, and there are no courteous state (means no vehicles yielded) at all. This is the same as what happens when the courtesy level is *zero* under uniform courtesy and no vehicles cooperate. A possible explanation is that although CDE and CDM have relatively high average courtesy levels, AV with very low courtesy level (on the left tail of the courtesy distribution) may sometimes result in system failure, where the courtesy proxy becomes very high (for both Egoism and Altruism) compared to RCL due to SV which attempt to change lane queueing up at the end of the segment. Under system failure, even AV with high courtesy levels would find it very costly or impossible to yield.

The mobility performance for CDM overall is better than CDE. This may be caused by the fact that the courtesy distribution of CDM is more skewed towards 1 compared to CDE (see Fig 6). Although under heavy traffic Egoism with CDM has smaller average speed for LC state than Egoism with CDE, this could be attributed to the randomness of simulation as more system failures occur for Egoism CDM (it is also hinted by the larger margin of standard deviation of simulation results).

Local Utilitarianism achieves the highest average mobility. This is an unsurprising result given that the courtesy strategy itself is encoded with a limited account of mobility maximization. At least in simple road networks we should expect that if individual interactions aim to maximize mobility, then the overall network will also maximize mobility.

**Fig 8:**
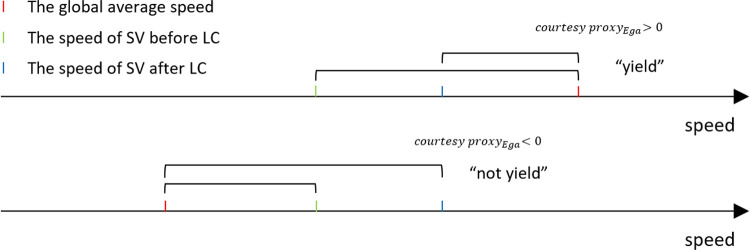
Different cooperative behavior under low and high global average speed in an egalitarianism system.

Local Maximin and Egalitarianism perform about the same under light and moderate traffic. It is worth noting that Egalitarianism degenerates sharply under heavy traffic. This is because in this case the cooperative behavior of AV is guided by the real-time global average speed (as in Fig 8). After changing lane, the speed of SV is assumed to reach the speed of TLV, which is usually an increase during the process. When the global speed is high, the system under Egalitarianism encourages (see Eq (11)) TLV to behave courteously because the resultant speed will become closer to global speed (the speed of TLV is assumed to be unchanged after LC). However, under heavy traffic when the global speed is typically lower, egalitarian principles might discourage TLV to yield to other vehicles because a local speed increase after changing lane would increase the variation of the system’s speed. Non-cooperation would further exacerbate the congestion situation and the global speed would decrease even more, finally leading to a system failure.

### 4.2 Safety

**Fig 9:**
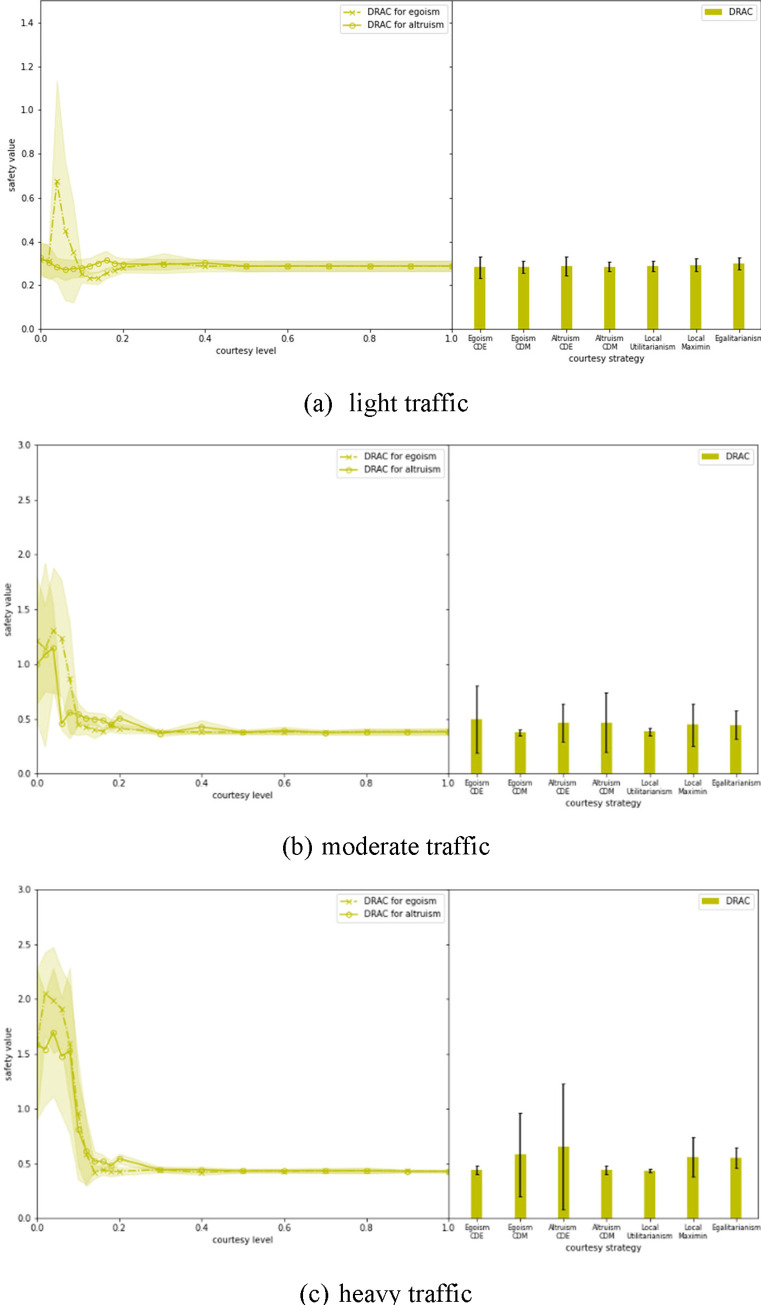
Evaluation of the safety performance of different courtesy strategies.

DRAC is a safety measure that mandates the least deceleration a TLV needs to execute for not colliding with a lane-changing SV. The smaller the DRAC is, the safer a yielding maneuver can be. In this study, each time a TLV yields to an SV, the corresponding DRAC is recorded and the average is shown in Fig 9. For Egoism and Altruism strategies with uniform courtesy, as the courtesy level increases, the DRAC first goes up and peaks at courtesy level = 0.04, then decreases and reaches the least value (less than 0.5 *m*/*s*^2^) at a relatively low courtesy level (about 0.1 for moderate traffic and 0.2 for heavy traffic). *This pattern is different from those seen in mobility results*, *where the system performance monotonically increases with courtesy level*.

Altruism is found to be safer than Egoism when a small amount of uniform courtesy in introduced into the system, but converges with Egoism at about 0.1 courtesy level under all three traffic inputs. It is proved again that variance (i.e., standard deviation of courtesy level) added to courtesy level could negatively affect the network performance especially under high traffic input. For instrumental courtesy strategies, Local Utilitarianism achieves the most stable (very small variance) and lowest DRACs.

### 4.3 Fairness

**Fig 10:**
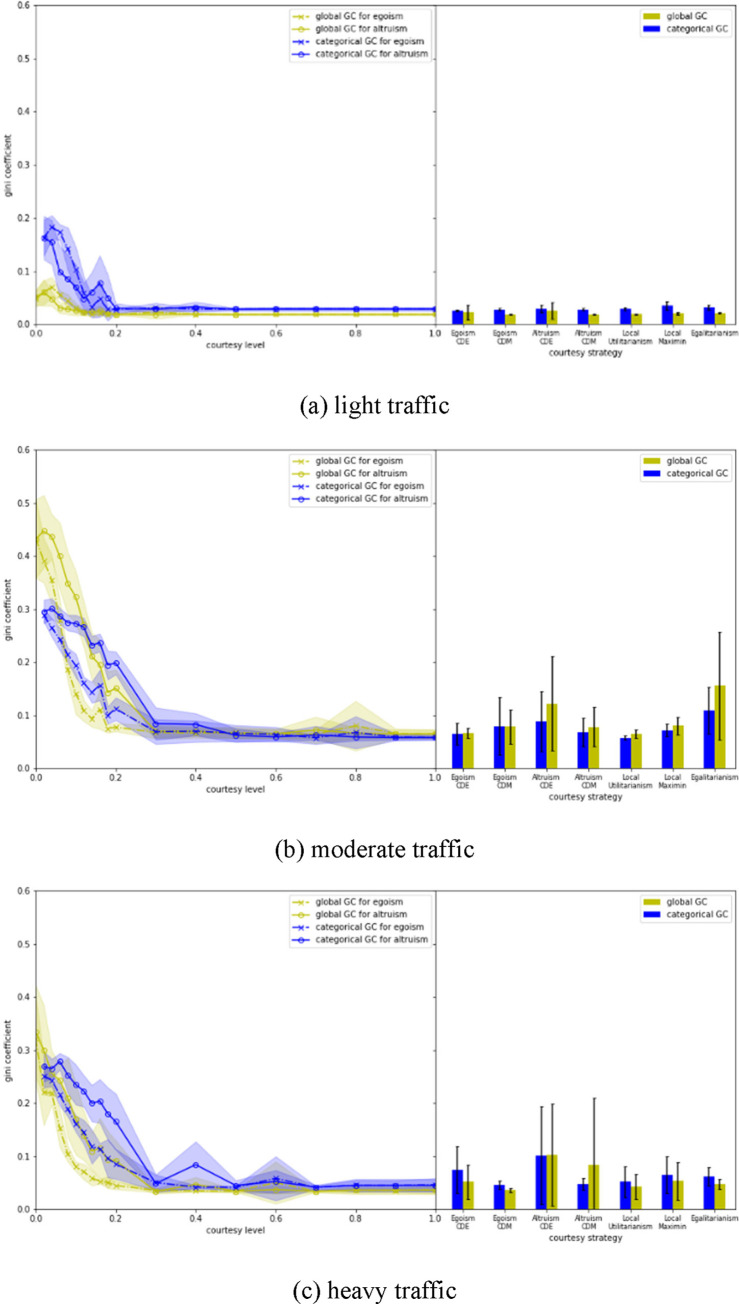
Evaluation of the fairness performance of different courtesy strategies.

An interesting pattern in the fairness evaluation results (Fig 10) is that a strategy with better mobility (for all states) often comes with better fairness. Egalitarianism under heavy traffic yields the lowest fairness performance, which is the opposite of its design intention. This probably is because it encourages courteous behavior that generates after-lane-change speed not surpassing the global average speed. When the global average speed is relatively low (usually under heavy traffic), cooperative behavior that can help to improve traffic operations and increase global speed would not be selected by the Egalitarianism strategy, although it works well under moderate and light traffic.

### 4.4 Vehicle state distribution

**Fig 11:**
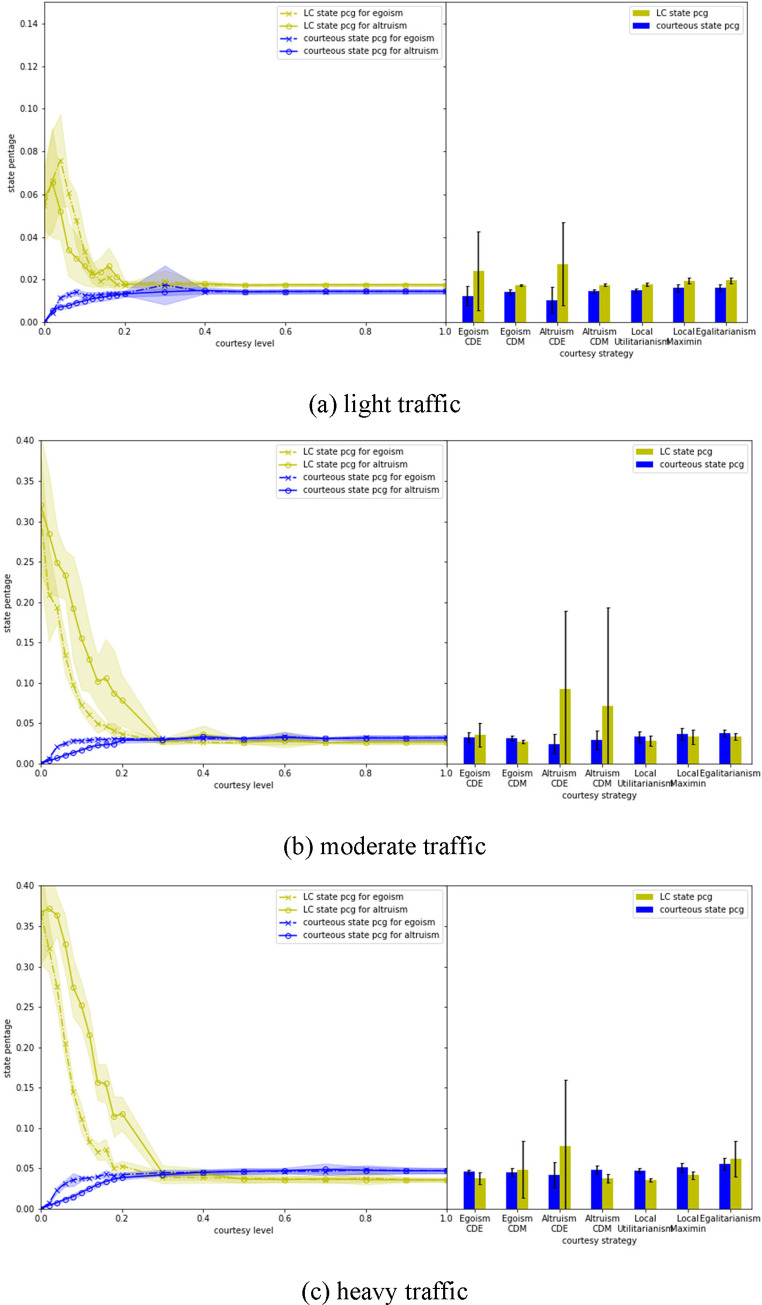
Vehicle state distributions for different courtesy strategies.

To better understand how each courtesy strategy affects system performance, the percentages of courteous state and lane-changing state generated by each strategy are computed and shown in Fig 11. Such percentages can reflect how often each courtesy strategy takes effects. Also, all courtesy strategies are evaluated using the same set of traffic demand (see Table 2). For each OD matrix, the *required* number of lane changes (i.e., to join/leave the highway) is the same for all strategies. Therefore, comparing the percentages of courteous and lane-changing states can be useful to understand how each courtesy strategy works, for example, how long vehicles are in the lane-changing process. When driving in a very courteous environment, the lane-changing state percentage (LCSP) should be lower than driving in an uncourteous environment where nobody yields, since it takes less time for vehicles to wait for safe gaps and change lanes.

It is worth noting that high courteous state percentage (CSP) does not necessarily result in low LCSP. Given a very congested on-ramp or work zone, courteous vehicles may need to wait for a long time for lane-changing vehicles to clear the bottleneck. In this case, both CSP and LCSP could be high.

**Fig 12:**
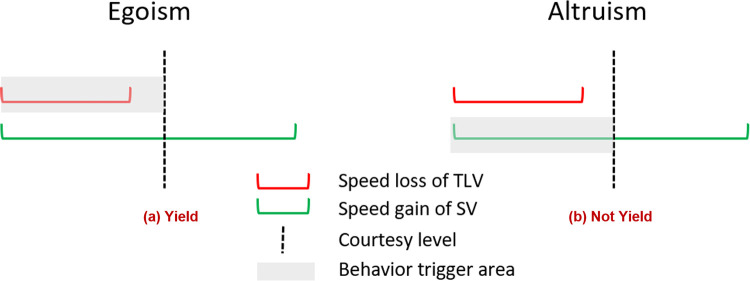
An illustration of the difference between egoism and altruism under the same uniform courtesy level.

Fig 11 clearly shows that with uniform courtesy between 0 and 0.3, Egoism has lower LCSP and higher CSP than Altruism under moderate and heavy traffic. Recall that Egoism also has better mobility results than Altruism in Fig 7 under the same traffic and courtesy conditions. This suggests that more courteous behaviors contribute to reducing the LCSP and improving system mobility performance. It also suggests the importance of carefully defining courtesy proxy and courtesy level. Egoism defines courtesy proxy from the perspective of TLV, while Altruism defines it based on SV. As the magnitude of courtesy proxy (i.e., speed difference) is different for TLV and SV (the speed gained by SV is usually larger than the speed lost by TLV) during lane change, courteous behavior would be more likely to occur under Egoism than Altruism (Since the CSP for Egoism is higher than Altruism as indicated in Fig 11) under the same uniform courtesy level. This explanation is further illustrated in Fig 12 and Eqs (4)–(7). For the same courtesy level and speed loss and gain, Egoism (Fig 12A) will yield based on Eqs (4)–(6), while Altruism (Fig 12B) will not based on Eq (7).

Another interesting finding from Figs 7 and 11 is that for a system to work well, the CSP should not be too low or too high. One example is the Egalitarianism. Low CSP indicates a lack of willingness for cooperation, which makes it difficult for SV (i.e., LC vehicles) to change lanes. While a very high CSP could be the result of a system failure.

### 4.5 Performance of individual segments

In addition to analyzing the performance of the entire network, the mobility results for individual segments and lanes are also collected. Table 4 shows the lane-by-lane average speed results for each of the four important segments.

**Table 4:** Lane-by-lane average speed under various courtesy strategies. (A, B, WZ, and C represent segment types; LL, ML and RL are for leftmost lane, middle lane, and rightmost lane, respectively; red and green colors are for congested and uncongested situations, respectively).

Courtesy Strategy	Lane Position	Light Traffic				Moderate Traffic				Heavy Traffic			
		A	B	WZ	C	A	B	WZ	C	A	B	WZ	C
Ego (Uniform Courtesy = 0)	LL	33.6	33.6	33.7	33.0	31.0	26.7	31.9	28.6	27.4	23.3	26.6	24.0
	ML	33.4	33.5	33.4	26.3	31.9	28.0	31.2	8.8	29.7	19.4	26.7	6.0
	RL	29.5	33.3	21.9	27.5	10.8	32.3	6.1	18.8	4.6	28.5	5.2	20.7
Ego (Uniform Courtesy = 0.5)	LL	33.7	33.7	33.7	33.2	33.6	33.6	33.6	32.1	33.4	33.6	32.6	29.6
	ML	33.3	33.6	33.3	33.2	32.3	33.1	31.9	32.1	30.9	31.8	28.8	29.3
	RL	32.8	33.4	31.6	28.9	31.1	33.3	29.5	29.4	29.6	33.1	27.2	28.7
Alt (Uniform Courtesy = 0)	LL	33.7	33.4	33.6	33.0	30.7	25.4	31.8	28.4	27.4	23.3	26.6	24.0
	ML	33.3	33.5	33.4	25.8	31.9	28.3	31.0	9.0	29.7	19.4	26.7	6.0
	RL	32.8	33.3	21.4	26.9	10.2	32.4	5.0	18.2	4.6	28.5	5.2	20.7
Alt (Uniform Courtesy = 0.5)	LL	33.7	33.7	33.7	33.2	33.6	33.6	33.6	32.1	33.5	33.6	32.8	29.8
	ML	33.3	33.6	33.3	33.3	32.3	33.1	31.9	32.1	30.9	31.8	28.9	29.5
	RL	32.8	33.4	31.6	29.0	31.1	33.3	29.4	29.8	29.5	33.1	27.3	28.9
Ego CDE	LL	33.7	33.6	33.7	33.2	33.6	33.6	33.6	30.9	33.5	33.6	32.8	29.7
	ML	33.3	33.6	33.3	31.6	32.3	32.9	31.9	30.4	30.9	32.0	29.1	29.3
	RL	32.4	33.4	30.9	28.4	31.0	33.3	28.6	27.9	29.5	33.1	27.4	28.4
Ego CDM	LL	33.7	33.7	33.7	33.2	33.5	33.6	33.6	31.8	33.4	33.5	32.6	29.1
	ML	33.3	33.6	33.3	33.3	32.3	33.1	31.9	31.9	30.9	31.4	29.2	27.5
	RL	32.8	33.4	31.6	29.1	31.0	33.3	29.5	28.7	29.6	33.0	25.4	28.2
Alt CDE	LL	33.7	33.5	33.7	33.2	32.3	33.5	33.5	31.0	33.5	33.4	31.9	27.9
	ML	33.3	33.6	33.3	31.9	32.2	32.9	32.0	25.9	31.0	30.7	28.5	24.0
	RL	31.2	33.3	30.3	27.8	25.5	33.3	25.7	26.7	27.4	32.9	23.0	26.3
Alt CDM	LL	33.7	33.7	33.7	33.2	33.5	30.9	32.9	31.1	33.4	33.6	32.7	29.1
	ML	33.3	33.6	33.2	33.3	32.3	32.5	31.6	28.9	30.9	32.0	26.0	29.3
	RL	32.8	33.4	31.6	29.1	28.7	33.2	26.5	26.5	29.5	33.1	27.4	27.8
LU	LL	33.7	33.7	33.7	33.2	33.6	33.6	33.6	31.5	33.5	33.6	32.8	29.8
	ML	33.3	33.6	33.2	33.2	32.3	33.1	31.6	31.6	30.9	31.8	28.8	29.9
	RL	32.8	33.4	31.5	28.8	31.1	33.3	29.4	28.5	29.5	33.1	27.2	28.5
LM	LL	33.7	33.6	33.6	33.1	33.6	33.3	33.5	30.6	32.4	32.5	32.0	29.3
	ML	33.3	33.5	33.2	33.2	32.2	32.6	31.8	30.7	30.5	30.9	28.2	29.5
	RL	32.7	33.2	31.0	28.7	30.1	32.8	29.1	27.4	28.9	31.5	25.5	27.9
Ega	LL	33.7	33.6	33.6	33.1	33.6	33.4	33.4	31.0	33.1	30.8	30.7	24.6
	ML	33.3	33.5	33.2	33.1	32.2	32.8	31.8	31.1	30.4	28.0	27.3	24.4
	RL	32.7	33.2	31.0	28.7	30.1	32.9	28.6	27.4	24.0	29.2	24.9	22.8

Under light traffic, the rightmost lane of the type C weaving area’s speed performance is among the lowest under all instrumental courtesy strategies, indicating this type C weaving area is the bottleneck of this corridor. For Egoism and Altruism with low uniform courtesy, it is hard for vehicles in the closed lane (i.e., rightmost lane) to merge in the work zone area, leading to low average speeds. Overall, the results suggest that Egoism and Altruism with low level of uniform courtesy do not work well under light traffic particularly for the type A and type C weaving areas and the work zone.

Similar trends could be found for moderate and heavy traffic. For Egoism and Altruism with uniform courtesy, a higher courtesy level is critical to accommodate the increased demand from lane changes. Congestion with low average speed is mostly found in the rightmost lanes of the type A weaving area and work zone and the middle lane of the type C weaving area. Results in Table 4 are consistent with the global average speed results in Fig 7. The low speed may be caused by few slow-moving vehicles seeking lane-changing opportunities due to a lack of cooperative TLVs.

**Fig 13:**
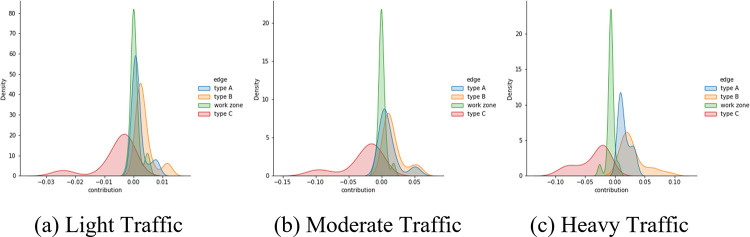
Contributions of each segment to the global average speed.

To further understand the contributions of each segment to the global mobility performance. The *contribution* of the *i*^*th*^ segment under each courtesy strategy is calculated using Eq (16).

$${CT}_{i}=\frac{v_{g}-v_{-i}}{v_{g}}$$ (16)

where *v*_*g*_ is the global average speed, and *v*_−*i*_ is the average speed without considering vehicles in the *i*^*th*^ segment. The idea behind Eq (16) is to calculate the removal effect, which tells the results difference between with and without the *i*^*th*^ segment. A positive *CT*_*i*_ value means having the *i*^*th*^ segment does not worsen the global performance, while a negative value suggests the opposite. The distributions of segment contribution are plotted in Fig 13. It is clear that the type C segment worsens the global average speed for most of the time. The contribution ranking (from negative to positive) based on mean *CT*_*i*_ is: type C, work zone, type A, and type B, and the contribution stability ranking (from highest to lowest) based on the variance of *CT*_*i*_ is: work zone, type A, type B, and type C.

## 5. Conclusions and discussion

Cooperation is a critical component of roadway traffic, particularly with the introduction of autonomous vehicles (AV). Previous studies often implicitly assume all AV to be fully cooperative, without considering the many possible strategies for implementing cooperation and the ethical and fairness issues involved. In this study, a systematic scheme to model and evaluate AV’s cooperative behavior is proposed. A total of five courtesy strategies are proposed. A modified rule-based AV behavior model CCM is presented and utilized to evaluate the proposed courtesy strategies.

SUMO simulation results at network and individual segment levels suggest that: (1) vehicles adopting courteous/cooperative behaviors could also benefit themselves particularly under heavy traffic; (2) for Egoism and Altruism, uniform courtesy is more effective than nonuniform courtesy with the same mean courtesy level; (3) variables such as traffic OD, courtesy level, and courtesy distribution (only applicable for non-instrumental strategies) play important roles in determining a system’s global performance. At the local level, segment type and lane position are additional parameters that affect traffic operations; (4) different performance metrics for the same courtesy strategy are correlated. For example, under moderate and heavy traffic Egoism has better mobility, worse safety, and better fairness performance than Altruism at the same level of uniform courtesy; and (5) Local Utilitarianism performs the best among all instrumental strategies in terms of mobility, safety, and fairness. Its performance is also very stable.

For future work, courtesy strategies following other principles can be explored. For example, an AV may yield with a probability, which can be either prespecified or dependent on real-time traffic conditions (e.g., how many times it has already yielded during that trip). AV may be able to earn credits for yielding to others and generating a net gain for the system. They can use such credits in the future to pay for other vehicles to yield to them. Also, in case multiple courtesy requests are received (although this does not happen often for lane changes on highways with two lanes in each direction), the request from the AV with the highest courtesy credit score will be selected to be served. Some of these ideas have already been explored in the context of intersection traffic management. For example, Dresner and Stone [30] proposed a First-Come-First-Serve (FCFS) strategy in their pioneer work on intersection traffic control with all autonomous vehicles. Carlino et al. [31] further extended the work by Dresner and Stone [30] and proposed an innovative aution-based autonomous intersection management strategy. Although there are important differences between lane change and intersection management, it would still be very interesting to modify the FCFS and auction-based methods for modeling courtesy in lane changes. When we put a price tag on courtesy, this may generate unwanted and complicated behavior if not properly handled. For example, a vehicle in the target lane may slow down on purpose to create a large/safe/attractive gap so that this gap can be sold at a high price. A possible but certainly not easy solution is that this vehicle pays to the system for the extra delay it has caused. Also, the lane-changing vehicle needs to determine the best gap to take given costs and the potential impacts on surrounding vehicles. These are all interesting but challenging problems to investigate and are beyond the scope of this paper.

In this study, the equations for LU, LM, and local Egalitarianism are not derived from rigorous mathematics and are essentially heuristic. Developing the optimal equations is a very challenging task both mathematically and philosophically. We will continue to investigate this important area in future research.

## References

[1] Committee S. O.-R. A. V. S. Taxonomy and Definitions for Terms Related to On-Road Motor Vehicle Automated Driving Systems. SAE Standard J, Vol. 3016, 2014, pp. 1–16.

[2] Wu C., Bayen A. M., and Mehta A. Stabilizing Traffic with Autonomous Vehicles. Presented at the 2018 IEEE International Conference on Robotics and Automation (ICRA), Brisbane, QLD, 2018.

[3] Schakel, W. J., B. van Arem, and B. D. Netten. Effects of Cooperative Adaptive Cruise Control on Traffic Flow Stability. Presented at the 2010 13th International IEEE Conference on Intelligent Transportation Systems—(ITSC 2010), Funchal, Madeira Island, Portugal, 2010.

[4] Milanés V., and Shladover S. E. Modeling Cooperative and Autonomous Adaptive Cruise Control Dynamic Responses Using Experimental Data. *Transportation Research Part C*: *Emerging Technologies*, Vol. 48, 2014, pp. 285–300. 10.1016/j.trc.2014.09.001.

[5] Desjardins C., and Chaib-draa B. Cooperative Adaptive Cruise Control: A Reinforcement Learning Approach. *IEEE Transactions on Intelligent Transportation Systems*, Vol. 12, No. 4, 2011, pp. 1248–1260. 10.1109/TITS.2011.2157145.

[6] Chu T., and Kalabić U. Model-Based Deep Reinforcement Learning for CACC in Mixed-Autonomy Vehicle Platoon. 2019.

[7] Turri V., Besselink B., and Johansson K. H. Cooperative Look-Ahead Control for Fuel-Efficient and Safe Heavy-Duty Vehicle Platooning. *IEEE Transactions on Control Systems Technology*, Vol. 25, No. 1, 2017, pp. 12–28. 10.1109/TCST.2016.2542044.

[8] Ren T., Xie Y., and Jiang L. New England Merge: A Novel Cooperative Merge Control Method for Improving Highway Work Zone Mobility and Safety. *Journal of Intelligent Transportation Systems*, Vol. 0, No. 0, 2020, pp. 1–15. 10.1080/15472450.2020.1822747.

[9] Wang, P., and C.-Y. Chan. Autonomous Ramp Merge Maneuver Based on Reinforcement Learning with Continuous Action Space. http://arxiv.org/abs/1803.09203. Accessed Aug. 21, 2022.

[10] Hegyi A., De Schutter B., and Hellendoorn H. Model Predictive Control for Optimal Coordination of Ramp Metering and Variable Speed Limits. *Transportation Research Part C*: *Emerging Technologies*, Vol. 13, No. 3, 2005, pp. 185–209. 10.1016/j.trc.2004.08.001.

[11] Milanes V., Godoy J., Villagra J., and Perez J. Automated On-Ramp Merging System for Congested Traffic Situations. *IEEE Transactions on Intelligent Transportation Systems*, Vol. 12, No. 2, 2011, pp. 500–508. 10.1109/TITS.2010.2096812.

[12] Schester, L., and L. E. Ortiz. Longitudinal Position Control for Highway On-Ramp Merging: A Multi-Agent Approach to Automated Driving. Presented at the 2019 IEEE Intelligent Transportation Systems Conference—ITSC, Auckland, New Zealand, 2019.

[13] Xie Y., Zhang H., Gartner N. H., and Arsava T. Collaborative Merging Strategy for Freeway Ramp Operations in a Connected and Autonomous Vehicles Environment. *Journal of Intelligent Transportation Systems*, Vol. 21, No. 2, 2017, pp. 136–147.

[14] Ren T., Xie Y., and Jiang L. Cooperative Highway Work Zone Merge Control Based on Reinforcement Learning in a Connected and Automated Environment. *Transportation Research Record*, 2020, p. 0361198120935873. 10.1177/0361198120935873.

[15] Shi, T., P. Wang, X. Cheng, C.-Y. Chan, and D. Huang. Driving Decision and Control for Automated Lane Change Behavior Based on Deep Reinforcement Learning. Presented at the 2019 IEEE Intelligent Transportation Systems Conference—ITSC, Auckland, New Zealand, 2019.

[16] Fridman L., Terwilliger J., and Jenik B. Deeptraffic: Crowdsourced Hyperparameter Tuning of Deep Reinforcement Learning Systems for Multi-Agent Dense Traffic Navigation. *arXiv preprint arXiv*:*1801*.*02805*, 2018.

[17] Oremus W. Tesla Insists Its Controversial Autopilot Software Is Saving Lives. Can It Convince the Rest of Us? Slate, Aug 04, 2016.

[18] Jiang L., Xie Y., Wen X., and Evans N. G. Cooperative Car-Following and Merging: A Novel Merge Control Strategy Considering Cooperative Adaptive Cruise Control and Courtesy. Presented at the Transportation Research Board, Washington D.C., 2021.

[19] Naus G. J., Vugts R. P., Ploeg J., van De Molengraft M. J., and Steinbuch M. String-S CACC Design and Experimental Validation: A Frequency-Domain Approach. *IEEE Transactions on vehicular technology*, Vol. 59, No. 9, 2010, pp. 4268–4279.

[20] Van Arem B., Van Driel C. J., and Visser R. The Impact of Cooperative Adaptive Cruise Control on Traffic-Flow Characteristics. *IEEE Transactions on intelligent transportation systems*, Vol. 7, No. 4, 2006, pp. 429–436.

[21] Powers T. M. Prospects for a Kantian Machine. *IEEE Intelligent Systems*, Vol. 21, No. 4, 2006, pp. 46–51. 10.1109/MIS.2006.77.

[22] Bonnefon J.-F., Shariff A., and Rahwan I. The Social Dilemma of Autonomous Vehicles. *Science*, Vol. 352, No. 6293, 2016, pp. 1573–1576. doi: 10.1126/science.aaf2654 27339987

[23] Leben D. A Rawlsian Algorithm for Autonomous Vehicles. Ethics and Information Technology, Vol. 19, No. 2, 2017, pp. 107–115. doi: 10.1007/s10676-017-9419-3

[24] De Moura N., Chatila R., Evans K., Chauvier S., and Dogan E. Ethical Decision Making for Autonomous Vehicles. 2020. 10.1007/s11948-020-00272-8 PMC7755871 33048325

[25] Bai W., Quan J., Fu L., Gan X., and Wang X. Online Fair Allocation in Autonomous Vehicle Sharing. Presented at the GLOBECOM 2017–2017 IEEE Global Communications Conference, 2017.

[26] Manual H. C. Highway Capacity Manual. *Washington*, *DC*, Vol. 2, 2000, p. 1.

[27] Lopez, P. A., M. Behrisch, L. Bieker-Walz, J. Erdmann, Y.-P. Flötteröd, R. Hilbrich, et al. Wagner, and E. Wiessner. Microscopic Traffic Simulation Using SUMO. Presented at the 2018 21st International Conference on Intelligent Transportation Systems (ITSC), 2018.

[28] Azath, M., R. S. D. Wahida Banu, and A. Neela Madheswari. Improving Fairness in Network Traffic by Controlling Congestion and Unresponsive Flows. Presented at the International Conference on Network Security and Applications, 2011.

[29] Cameron M. W. EFFICIENCY AND FAIRNESS ON THE ROAD: STRATEGIES FOR UNSNARLING TRAFFIC IN SOUTHERN CALIFORNIA. 1994.

[30] Dresner K., and Stone P. A Multiagent Approach to Autonomous Intersection Management. *Journal of artificial intelligence research*, Vol. 31, 2008, pp. 591–656.

[31] Carlino, D., S. D. Boyles, and P. Stone. Auction-Based Autonomous Intersection Management. Presented at the 16th International IEEE Conference on Intelligent Transportation Systems (ITSC 2013), 2013.

